# Improved predictive formulae for wave overtopping at sloped breakwaters using interpretable machine learning models

**DOI:** 10.1371/journal.pone.0337830

**Published:** 2025-12-10

**Authors:** M. A. Habib, S. Abolfathi, J. J. O’Sullivan, M. Salauddin

**Affiliations:** 1 UCD Dooge Centre for Water Resources Research, UCD School of Civil Engineering, and UCD Earth Institute, University College Dublin, Dublin, Ireland; 2 School of Engineering, University of Warwick, Coventry, United Kingdom; University of Hamburg: Universitat Hamburg, GERMANY

## Abstract

Accurate prediction of mean wave overtopping discharge is essential for the safe and cost-effective design of coastal defence structures. While traditional empirical, physical, and numerical models remain important, Machine Learning (ML) has recently emerged as a powerful complementary tool. This study presents a ML–based framework to predict mean wave overtopping discharge at sloped breakwaters, with a focus on both predictive accuracy and model interpretability, supported by a series of structured pre- and post-processing steps. Five ML algorithms were evaluated: two decision tree–based models, i.e., Random Forest (RF) and Gradient Boosted Decision Trees (GBDT), and three kernel-based models, i.e., Artificial Neural Networks (ANN), Support Vector Regression (SVR), and Gaussian Process Regression (GPR). The models were trained and validated using the EurOtop (2018) dataset on sloped breakwaters. Among them, GPR yielded the best predictive performance, achieving an R² of 0.80 and the lowest RMSE, MAE, and RAE values (0.100, 0.013, and 0.30, respectively), indicating a strong agreement with observed data. Feature importance analysis revealed that Relative Freeboard and Freeboard Deficit (FD) were the most influential parameters across the models. To enhance interpretability and practical usability, we translated the ML findings into mathematical expressions using polynomial regression and Genetic Programming (GP). A new set of simplified equations was developed to estimate mean overtopping discharge (*q*) based solely on FD, effectively modelling the relationship between FD and ln(*q*) within the EurOtop dataset. The proposed formulae provide coastal engineers with a rapid, interpretable, and reliable tool for estimating mean wave overtopping, significantly enhancing design efficiency and decision-making under uncertainty. By bridging the gap between advanced data-driven techniques and practical engineering needs, this work advances the integration of ML into coastal infrastructure design and supports the development of more adaptive and climate-resilient defence systems.

## 1.0 Introduction

**Fig 1:**
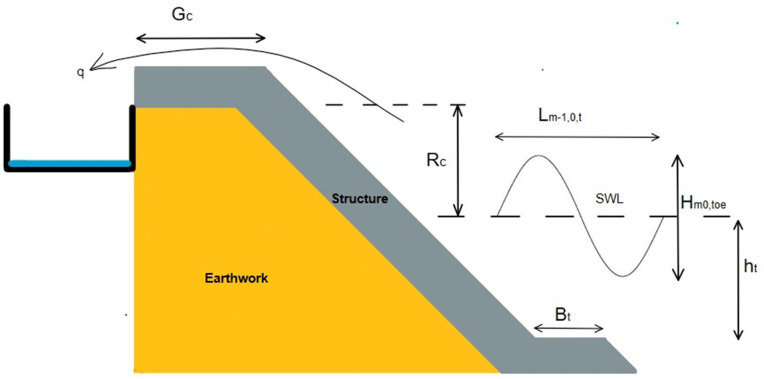
Schematic of overtopping at sloping structures.

Coastal overtopping is a term that refers to the phenomenon where waves breach coastal defence structures under the influence of extreme storm surges, leading to the inundation of hinterland areas. Coastal defence structures, such as simple sloping dykes (**Fig 1**), are constructed to prevent overtopping in designated coastal zones. Thus, their design must be precise and grounded in detailed understanding of the wave-structure interactions, to ensure acceptable level of protection. A critical parameter in the design of these structures is the wave overtopping rate, denoted by ‘*q*’ which represents the discharge per meter width of the structure and is typically measured in m^3^/s/m or l/s/m. Wave overtopping discharge is influenced by various factors, including local wave conditions (such as wave height, wave period and water depth) and the geometrical characteristics of the coastal defence structures [1–5]. Recent studies suggests that the frequency of overtopping events is likely to increase due to the combined effects of natural and anthropogenic factors, including climate change, urbanization, and coastal tourism. Previous climate vulnerability studies (e.g.,[6] [7]) highlighted the compounded impacts of climate change, such as coastal flooding, and the uncertainty surrounding flood resilience. Therefore, a precise approach to estimating overtopping discharge is essential to enhance the resilience of coastal infrastructure and mitigate coastal hazards effectively.

The semi-empirical models prescribed by [7] provide a comprehensive framework for estimating mean overtopping discharge. However, other approaches, including physical experiments, numerical analysis, and ML applications, are increasingly prominent in recent literature. This study explores the performance and interpretability of two distinct classes of ML algorithms: kernel-based methods and decision tree (DT)–based models. These algorithms are trained and validated using the experimental dataset from [7] to enable a systematic comparison of their predictive capabilities. Beyond predictive performance, the study emphasizes model interpretability by assessing physical consistency and translating ML insights into practical engineering tools. Specifically, mathematical expressions derived from the trained models are proposed to support design applications. The research is motivated by two key gaps in the existing literature: the limited interpretability of ML models applied to wave overtopping prediction and the lack of simplified, design-ready tools derived from such models. This study addresses critical gaps in current wave overtopping research through two main objectives. First, it aims to develop a (ML) framework for predicting mean wave overtopping discharge at simple sloped coastal structures. To this end, three kernel-based algorithms, including Artificial Neural Networks (ANN), Support Vector Regression (SVR), and Gaussian Process Regression (GPR), were employed alongside two decision tree–based (DT) models: Random Forest (RF) and Gradient Boosted Decision Trees (GBDT). Advanced ML techniques, including feature selection and feature importance analysis, were incorporated to enhance model accuracy and interpretability. The second objective focuses on translating ML model outputs into physically meaningful and practically usable equations. A unified mathematical formulation was derived using Genetic Programming (GP), an evolutionary algorithm, based on the most influential parameters identified through ML analysis. These equations capture the underlying relationships governing overtopping processes and provide coastal engineers with an efficient, design-oriented tool for preliminary estimation of mean overtopping discharge. This dual approach bridges the gap between black-box ML models and practical engineering design, offering a transparent and scalable methodology for wave overtopping assessment.

## 2.0 Related work

A review of the state-of-the-art literature on the applications of ML in wave overtopping prediction for coastal defence structures reveals that issues of interpretability and modelling insights remain areas for further investigation [8]. Recent studies [8,9] have utilised Evolutionary Polynomial Regression (EPR) algorithms, such as (GP, to develop or improve equations originally derived from experimental and numerical studies. These studies underscore the need to complement ML algorithms with tools, such as predictive equations, to enhance understanding of their contribution to overtopping prediction in terms of efficacy and simplicity. Existing research on overtopping estimation has focused on various aspects, including prediction, modelling, mitigation strategies, and the impacts of overtopping on coastal structures. Previous studies have examined the factors contributing to overtopping events in different parts of the world [10], the spatial distribution of overtopping waves at coastal structures, [11], and the effectiveness of retrofitting techniques, such as recurve walls, in mitigating overtopping [4]. Sustainability-oriented measures, such as eco-engineering, have also been investigated for their potential to reduce overtopping events [12,13]. In addition to small-scale laboratory tests, numerical simulations have led to the developments of new physics-based design formulae for predicting overtopping discharge at vertical walls and validating the accuracy of numerical models in overtopping estimating [14,15].

The EurOtop manual [7] offers the most extensive collection of empirical formulas for estimating overtopping across various coastal defence structures, serving as a global design guide. This manual’s open-source dataset, consisting of over 18,000 tests, draws from field observations and laboratory studies on wave overtopping and its effects on coastal structures. Other notable contributions to empirical wave overtopping estimation include semi-empirical formulas by [16,17].

For design and assessment purposes, [18,19] proposed a set of equations, i.e., Eq (1) and Eq (2), to estimate the mean overtopping discharge at smooth sloping structures under breaking and non-breaking wave conditions. These equations have since been adopted in [7] and are widely utilized in contemporary coastal engineering practice.

(For breaking waves: $\xi_{m-\text{1},\text{0}}$ < 2);


$$\frac{q}{\sqrt{gH_{m\text{0}}^{\text{3}}}}=\;\frac{\text{0}.\text{0}\text{2}\text{3}}{\sqrt{tan\alpha }}{\;\xi }_{m-\text{1},\text{0}}\;\text{exp}\left[{-\left(\text{2}.\text{7}\frac{R_{c}}{\xi_{m-\text{1},\text{0}}H_{m\text{0}}\gamma_{f}}\right)}^{\text{1}.\text{3}}\right]$$ (1)

(For non-breaking waves: $\xi_{m-\text{1},\text{0}}$ > 2);


$$\frac{q}{\sqrt{gH_{m\text{0}}^{\text{3}}}}=\;\text{0}.\text{0}\text{9}\;\text{exp}\left[{-\left(\text{1}.\text{5}\frac{R_{c}}{H_{m\text{0}}\gamma_{f}}\right)}^{\text{1}.\text{3}}\right]$$ (2)

where q is the mean overtopping discharge, $H_{m\text{0}}$ denotes the significant wave height, $R_{c}$ is the crest-freeboard, $\gamma_{f}$ is the influence factor for permeability and roughness of the slope, and $\xi_{m-\text{1},\text{0}}$ is a wave breaker parameter calculated from Eq (3):


$$\xi_{m-\text{1},\text{0}}=\;\frac{\text{tan}\alpha }{\sqrt{\frac{{Hm}_{\text{0}}}{L_{m-\text{1},\text{0}}}}}$$ (3)

where $L_{m-\text{1},\text{0}}$ is the wavelength in deep water based on the spectral time period $T_{m-\text{1},\text{0}}(=\frac{gT_{m-\text{1},\text{0}}^{\text{2}}}{\text{2}\pi })$.

Advances in computational power and resources have encouraged the application of ML algorithms in wave overtopping estimations [18,19]. The EurOtop manual [7] has endorsed the application of ANN in the overtopping prediction. A review [20] revealed that ML algorithms are widely used to estimate wave overtopping at coastal structures with varying geometrical configurations. The study identified kernel-based algorithms such as ANN and SVR and tree-based algorithms such as RF and GBDT are popular choices for overtopping estimation.

A recent work by [21] employed deep learning techniques, specifically neural networks, to assess wave overtopping within a port setting. The study in [18,19] demonstrated the effectiveness of GBDT models in reducing prediction errors and enhancing the precision of wave overtopping discharge estimates, highlighting the potential of ML to improve coastal engineering predictions. Also, [22] explored the use of ANN-based models, including multilayer perceptron (MPNN) and general regression neural networks (GRNN), as well as support vector machines (SVM), to predict wave overtopping at coastal structures with straight slopes. The study of [23] conducted model tests to address knowledge gaps in wave overtopping for step revetments and developed new empirical formulas to enhance prediction accuracy for both breaking and non-breaking waves. Similarly, [24] investigated various ML techniques, including RF, GBDT, SVR, and ANN, concluding that RF provided the most accurate predictions for wave overtopping at vertical seawalls. The work in [25] presented models using GBDT to predict mean overtopping discharge. Additionally, [26] emphasized the transformative impact of deep learning on artificial intelligence, proposing a convolutional neural network (CNN) model to predict wave overtopping under diverse conditions. The study in [27] applied eight linear and nonlinear ML models to the same dataset, developing a pipeline to select the optimal model for specific overtopping scenarios. Other notable studies, such as those by [28] and [29], have focused on developing ANN models to estimate wave reflection and overtopping discharge, further demonstrating the versatility and efficacy of ML approaches in this field. Integrating ML with traditional empirical formulas and numerical models offers a promising approach to enhancing the accuracy and reliability of coastal wave overtopping estimates. By incorporating advanced computing methods, researchers and coastal engineers can achieve rapid assessments of wave overtopping discharge and volumes. Additionally, pre-processing, and post-processing tools, such as feature selection and feature importance analysis, strengthen data analysis, enabling the development of robust predictive models to mitigate wave-induced hazards effectively.

In [9], the authors employed EPR to derive an equation for estimating mean overtopping rates at smooth dikes and vertical walls. Using a composite dataset from EurOtop and other experiments, they developed formulas involving 3–4 structural and hydraulic parameters for overtopping prediction. The study introduced the concept of *freeboard deficit* (FD), calculated using Eq (4), to account for wave run-up assessments, including wave period and local water depth. According to the authors, FD provided a more explicit description of the overtopping phenomenon than relative freeboard.


$$FD=\text{1}-\;\frac{R_{c}}{{Ru}_{max}}$$ (4)

where $R_{c}$ is the crest freeboard and ${Ru}_{max}$ is the maximum run-up of all waves in sea state and is calculated from the run-up level exceeded by 2% of the incident waves $R_{u\text{2}\%}$ [30]:


$$\frac{R_{u\text{2}\%}}{H_{mo}}=\text{4}.\text{3}-\;\frac{\text{1}.\text{6}}{\sqrt{\xi_{m-\text{1},\text{0}}}}$$ (5)

$${Ru}_{max}=\text{1}.\text{5}\text{4}R_{u\text{2}\%}$$ (6)

where $H_{mo}$ is the significant wave height and $\xi_{m-\text{1},\text{0}}$ is wave breaking parameter.

The effectiveness of EPR was further demonstrated in the study by [8], which explored the impact of crown walls and bullnoses on reducing mean overtopping rates at dikes. The authors argued that EPR algorithms offer an advantage over conventional methods by providing interpretable mathematical formulas that can be analysed and refined. Using both numerical and experimental data, the study employed the Genetic Programming module of EPR to develop an equation representing mean overtopping rates at dikes with crown walls and bullnoses. The two studies discussed above focused on developing interpretable equations using EPRs from experimental and numerical data. While these equations benefited from EPR-based refinements, the development of equations derived specifically from ML model insights remains limited. This study addresses this gap by applying Genetic Programming (GP), an EPR technique, to translate ML model insights into mathematical terms, aiming to create a simplified, single-variable equation for the rapid preliminary assessment of mean wave overtopping rates at sloped structures. In this work, three kernel-based and two decision tree-based ML algorithms are employed to obtain a comprehensive view of influential parameters for predicting overtopping at simple sloped structures. Modern pre-processing and post-processing methods—including feature selection, hyperparameter tuning, and feature importance analysis (SHAP)—enhance the interpretability of the models. Based on the feature importance findings, GP is used to derive equations involving a single parameter, *freeboard deficit* (FD), to estimate overtopping across varied wave conditions. This study, therefore, provides a direct mathematical interpretation of key insights from multiple ML algorithms. The novel contribution of this study lies in the development of straightforward, design-oriented tools derived from the insights of ML–based overtopping prediction models. These tools are grounded in a comprehensive benchmarking of multiple ML algorithms, ensuring both predictive robustness and practical applicability for coastal engineering design.

## 3.0 Materials and methods

### 3.1 Background on machine learning algorithms for wave overtopping modelling

#### 3.1.1 Decision trees.

Decision Trees (DTs) are fundamental components of tree-based ML algorithms, capable of addressing both classification and regression problems [31]. DTs are easy to understand and interpret as they break down complex tasks into a series of straightforward, hierarchical steps. The structure of a DT resembles a tree, with nodes organized from the root to the leaves [32,33]. Training a DT model involves recursive splitting and multiple regression steps, starting from the root node and continuing until a specified stopping criterion is met [34]. Each leaf node in a DT can be theoretically approximated as a simple linear regression model. Subsequently, a pruning process reduces model structural complexity to improve generalization. RF and GBDT are two powerful DT-based ML algorithms widely used in regression tasks, each built upon an ensemble of DTs [35,36]. RF has shown high predictive accuracy on high-dimensional datasets, as demonstrated by [37]. RF’s effectiveness in coastal overtopping estimation has been explored in studies such as those by [38–40]. The RF algorithm generates predictions by averaging the outputs from all DTs in the ensemble, with its predictive model summarized by Eq (7) [34]:


$$\begin{array}{c} {\hat{f}}_{rf(x)}^{K}=\frac{\text{1}}{K}\sum {\mathrm{\backslash nolimits}}_{k=\text{1}}^{K}T(x) \end{array}$$ (7)

where ${\hat{f}}_{rf(x)}^{K}$ is the average predicted quantity from the RF function rf(x) with x input vectors constituted from the features in a data set and K is number of Decision Trees, T(x) in the ensemble. The RF algorithm is designed to reduce overfitting and improve generalization by optimizing the exposure to the training data by a technique known as bagging (see Fig 3). Bagging enhances the robustness of the input data by generating multiple subsets of the training dataset through random sampling with replacement. This process increases prediction accuracy while also improving model stability. Furthermore, RF’s ability to handle missing data makes it particularly useful for complex prediction tasks.

**Fig 2:**
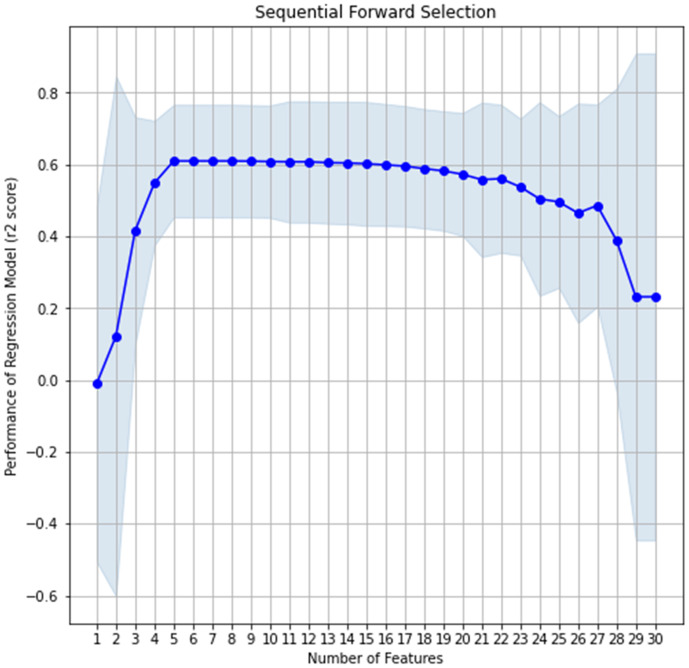
Extraction of the number of impactful features using sequential forward selection.

Gradient Boosted Decision Tree (GBDT) is an ensemble-based algorithm widely utilized for classification and regression tasks in data science [41]. GBDT employs the gradient boosting technique, which is particularly effective for handling nonlinear data and complex regression tasks [42]. Gradient boosting iteratively computes the Mean Squared Error (MSE) between predicted and actual values, converting it into a loss function. This loss function is then minimized using gradient descent, refining the model’s predictive accuracy at each step. Like RF, GBDT is highly effective for pattern recognition in high-dimensional datasets characterised by intricate non-linear relationships [43].

The weighted ensemble output of decision trees is governed by Eq (8):


$$\begin{array}{c} \hat{y}(x)=\;\sum {\mathrm{\backslash nolimits}}_{t}w_{t}h_{t}(x) \end{array}$$ (8)

where $w_{t}$ and $h_{t}(x)$ are the weight and output of an individual tree in the ensemble.

Following the outputs from the ensemble of decision trees, the optimization process refines the model through an objective function F(x), as shown in Eq (9). This function comprises two key components: a loss component, which measures the prediction error, and a regularization component, which prevents overfitting and enhances model generalization.


$$\begin{array}{c} F(x)=\;\sum {\mathrm{\backslash nolimits}}_{i}l({\hat{y}}_{i},y_{i})+\;\sum {\mathrm{\backslash nolimits}}_{t}\Omega (f_{t}) \end{array}$$ (9)

where $l({\hat{y}}_{i},y_{i})$ is the loss component after *‘i’* number of iterations; ${\hat{y}}_{i},y_{i}$ are predicted and actual values, respectively, and $\Omega (f_{t})$ is the regularization component of tree ‘*t’* which depends on the structural complexity of individual trees. The GBDT algorithm works to minimize the loss function shown in Eq 11.

### 3.1.2 Artificial neural network

Artificial Neural Networks (ANNs) are computational models inspired by the functionality of the human brain. They consist of interconnected artificial neurons designed to process and analyse data by mimicking the way biological neurons communicate. ANNs are trained on input data to recognize patterns, learn relationships, and make predictions or decisions based on the learned information [44]. Due to their adaptability and ability to model complex non-linear systems, ANNs have found applications across various fields, including medicine, economics, engineering, and environmental sciences [45]. In the training process, ANN iteratively adjust their internal parameters (weights and biases) using input data and error feedback to improve performance. This training enables the network to generate accurate outputs for unseen data [46].

ANNs have proven to be highly effective in predicting wave overtopping at coastal structures. For example, studies have demonstrated their ability to estimate wave overtopping discharge quantities with remarkable accuracy using experimental datasets such as the CLASH database [28]. Additionally, ANN have been adopted to predict wave transmission and reflection coefficients. Their capability to handle large datasets, adapt to specific data characteristics, and deliver rapid results makes them invaluable in artificial intelligence applications for coastal engineering [24,29,39,47,48].

In this study, a feed-forward, back-propagation Multi-Layer Perceptron (MLP) ANN was employed, and this type of ANN architecture is particularly suited for regression tasks, making it an ideal choice for predicting overtopping rates. The feed-forward neural network (FFNN), as detailed by [49] is a type of ANN where the information flows in a single direction, from the input layer through one or more hidden layers to the output layer, without looping back. This structure ensures a straightforward flow of data and computations, making FFNNs well-suited for tasks involving prediction and classification.

### 3.1.3 Support vector regression

SVR is a supervised ML algorithm designed specifically for regression tasks [50]. Known for its robustness and versatility, SVR is particularly effective for addressing nonlinear relationships in datasets ranging from small to large scales [51]. Its strength lies in its ability to balance complexity and generalization, making it an ideal choice for tasks where precision and adaptability are crucial. SVR is based on the Structural Risk Minimization (SRM) principle, which aims to minimize the upper bound of generalization errors. Unlike traditional empirical risk minimization, which only focuses on reducing training errors, SRM considers both the training error and the model’s confidence interval. This dual focus allows SVR to achieve superior performance in avoiding overfitting and underfitting, making it highly effective for predictive modelling tasks [32].

A defining feature of SVR is its use of kernel functions to transform input data into a higher-dimensional feature space, enabling the algorithm to handle nonlinear relationships in the data effectively. Commonly used kernel functions include linear, polynomial, radial basis function (RBF), and sigmoid kernels, each suited to distinct types of datasets and complexities [52]. Once data is mapped into the feature space, SVR performs regression by finding a hyperplane that minimizes errors within a defined margin, ensuring model simplicity while maintaining accuracy. The SVR objective function can be mathematically expressed as shown in Eq (10), which represents the optimization process employed to minimize prediction errors while maximizing the margin between the predicted and actual values.


$$\begin{array}{c} f(x)=\;\sum {\mathrm{\backslash nolimits}}_{i}^{l}y_{i}\left(\partial_{i}-\partial_{i}^{*}\right)K\left(x_{i},x\right) \end{array}$$ (10)

where $K\left(x_{i},x\right)$ is the kernel function, l denotes the number of input data features, and $\partial_{i},$ $\partial_{i}^{*}$ are Lagrangian multipliers. In this study, Gaussian Radial Basis Function (RBF) was adopted as the kernel function The ability of a Gaussian RBF to tackle non-linear and high dimensional data sets is reported in the previous studies [53,54].

### 3.1.4 Gaussian process regression

GPR is a non-parametric ML method widely used for regression tasks, particularly when modelling complex, nonlinear relationships. Unlike parametric models, GPR does not assume a specific functional form for the data, making it highly adaptable to various problem domains. Instead, it leverages kernel functions to estimate characteristics of unknown functions, such as maximum and minimum values, while maintaining flexibility and precision [55].

GPR is especially effective for analysing high-dimensional and complex datasets by mapping input vectors to corresponding output vectors. For an input vector $x\in R^{d}$, in a *d*-dimensional space and an output vector y∈R, GPR maps the relationship between inputs and outputs as a Gaussian process. This mapping allows the regression to estimate the distribution of outputs given a new set of inputs, enabling probabilistic predictions. A key feature of GPR is its ability to model the covariance between predicted and actual values. In overtopping estimation, [27] describe GPR as a framework where the covariance function quantifies the relationship between the observed overtopping discharge and the residual difference between actual and estimated quantities. This covariance is modelled using kernel functions, such as radial basis functions (RBF) or polynomial kernels, which define the smoothness and generalization properties of the regression.

### 3.1.5 Genetic programming

Genetic programming (GP) is a biologically inspired ML algorithm that derives advanced regression by learning from empirical data. Unlike traditional regression, GP uses symbolic regression to identify mathematical relationships between variables and deduce interpretable equations that model physical processes, such as wave overtopping. This capability makes it a valuable tool for deriving empirical formulas for complex environmental systems [8]. Symbolic regression is distinct from conventional regression in that it automatically determines the optimal form of the regression equation. The GP algorithm achieves this by employing a library of analytical elements, such as power, square root, exponential, hyperbolic, and trigonometric functions, to fit an appropriate mapping function between input variables. This approach enables the discovery of equations tailored to specific datasets and physical phenomena. The Genetic Programming (GP) algorithm begins by generating an initial “first population” of candidate mathematical formulas (referred to as “individuals”) based on a user-defined set of variables or “genes.” Each formula combines these variables using analytical functions, such as powers, square roots, exponentials, or trigonometric operations, as discussed earlier. This process aims to explore a wide range of potential solutions for the regression problem. The generated formulas are then iteratively “mutated” over successive “generations” until the optimal formula is derived—that is, the formula that best maps the selected variables. At each generation, the accuracy of the formulas is evaluated through training and testing, and this information is used to refine the next generation. The process mimics natural evolution through three fundamental steps: reproduction, mutation, and crossover. The output of a GP algorithm is a regression function consisting of several parameters (genes) that represent a specific process. For instance, in the context of overtopping, these parameters would be expressed in a mathematical equation to predict overtopping quantities. Such an equation typically comprises weighted parameters and a bias term and can be represented as shown in Eq (11):


$$y=\;x_{\text{0}}+\;w_{\text{1}}x_{\text{1}}+\;w_{\text{2}}x_{\text{2}}+\ldots +\;w_{n}x_{n}$$ (11)

where y is the output term, $x_{\text{0}}$ denotes the bias and $w_{\text{1}}x_{\text{1}}\;$ to $w_{n}x_{n}$ are the weighted input parameters. The weights are calculated as regression coefficients during each iteration by the algorithm.

### 3.2 Feature selection and feature transformation

High dimensional datasets, such as the overtopping dataset, can induce data redundancy, which may negatively impact the robustness of ML-based prediction models. Additionally, redundant data can lead to increased computational costs and longer processing times. Feature selection techniques can be implemented to address data redundancy by extracting a meaningful subset of features that most effectively represent the process or phenomenon under investigation [56]. Typically, feature selection involves permutation-combination and statistical analysis. During the permutation-combination process, subsets of features are iteratively selected, and their correlation with the target variable is assessed through regression analysis. Features are ranked based on their statistical significance to the target variable, enabling the extraction of the most impactful features from a high-dimensional dataset.

While feature selection identifies the number of meaningful features, feature transformation techniques provide deeper insights by pinpointing the specific impactful features. One of the most widely used feature transformation techniques is Principal Component Analysis (PCA). PCA reduces the variation in large datasets by identifying and transforming the data into uncorrelated principal components that capture the maximum variance in the dataset without significant information loss [50,57].

In this study, feature selection and feature transformation techniques were coupled to extract the most impactful features from the overtopping dataset. The Forward Sequence Feature Selection (FSFS) method was adopted for feature selection. The FSFS method initiates from an empty set, iteratively adding features from the dataset and conducting linear regression to deduce the impact of the selected features. Cross-Validation (CV) score is calculated for each combination of features, and the feature set that maximises the CV score is identified as optimal. As shown in Fig 2, the regression performance for the overtopping dataset decreased after the addition of the 19^th^ feature. This observation indicates that the optimal number of features is 19. PCA was subsequently applied to validate these results, revealing that 19 principal components explained the majority of variance in the dataset. Therefore, it could be determined that 19 features, namely, *H*_*m0,d*_, *T*_*p,d*_, *h, H*_*m0,toe*_, *T*_*m,toe*_, *h*_*t*_, *cotα*_*d*_, *cotα*_*u*_, *D*_*50,d*_, *D*_*50,u*_, *R*_*c*_, *B,* γ_f_, *tanα*_*B*_, *G*_*c*_, *RF, CF, FD and Rc/H*_*m0,toe*_ (see Table 2) are the most impactful as input features in the ML models to estimate the mean overtopping rate.

**Table 1:** Overview of hyperparameters optimisation.

Algorithm	Source code of hyperparameters	Best values
SVMR	{‘kernel’: (‘linear’, ‘rbf’,’poly’), ‘C’:[5, 10, 15, 20],’gamma’: [‘auto’,’scale’],’epsilon’:[0.1,0.2,0.5,0.3,0.4]}	‘kernel’: ‘rbf; ‘gamma’: ‘auto;’ ‘epsilon’: 0.1; ‘C’: 20
RF	{‘n_estimators’: np.arange(50, 500, 10), ‘max_depth’: np.arange(3,12,3), ‘min_samples_split’: np.arange(2, 3), ‘min_samples_leaf’: np.arange(2, 3)}	n_estimators’: 60, ‘ max_depth’ : 3, ‘ min_samples_split’ : 2, ‘min_samples_leaf: 3,
GBDT	{‘max_depth’: [3, 5, 6, 10, 15, 20], ‘learning_rate’: [0.01, 0.1, 0.2, 0.3], ‘subsample’: np.arange(0.5, 1.0, 0.1), ‘colsample_bytree’: np.arange(0.4, 1.0, 0.1), ‘colsample_bylevel’: np.arange(0.4, 1.0, 0.1), ‘n_estimators’: [100, 500, 1000]}	{‘subsample’: 0.90, ‘n_estimators’: 100, ‘max_depth’: 15, ‘learning_rate’: 0.3, ‘colsample_bytree’: 0.4, ‘colsample_bylevel’: 0.5}
ANN	{ ‘hidden_layer_sizes’: [(150,100,50), (120,80,40), (100,50,30)], ‘max_iter’: np.arange(50,500,50), ‘activation’: [‘logistic’,’tanh’, ‘relu’], ‘solver’: [‘sgd’, ‘adam’], ‘alpha’: [0.0001, 0.0005], ‘learning_rate’: [‘constant’,’adaptive’], ‘early_stopping’: [False] }	{‘activation’: ‘relu’, ‘alpha’: 0.0005, ‘early_stopping’: False, ‘hidden_layer_sizes’: (120, 80, 40), ‘learning_rate’: ‘constant’, ‘max_iter’: 200, ‘solver’: ‘adam’}
GPR	{ ‘kernel’: [C(1.0, (1e-3, 1e3)) * RBF(length_scale=1.0), C(1.0, (1e-3, 1e3)) * RBF(length_scale=0.1), C(1.0, (1e-3, 1e3)) * RBF(length_scale=10.0)], ‘alpha’: [1e-10, 1e-5, 1e-3, 0.1, 1.0, 10.0]	‘Kernel’:C(1.0, (1e-3, 1e3)) * RBF(length_scale=0.1); ‘alpha’:1e-5

**Table 2:** Filtration criteria of different overtopping parameters to extract data for simple sloped structures.

Filtration criteria	Remarks
H_m0,toe_ ≤ 0.5	to include entries for small-scale records
γ_f _< 0.6; except γ_f _= 0.55	to include permeable sloped walls
1.33 ≤ cot α ≤ 2	to include walls with mild slope
B = 0	to include walls with no Berm
cot α_u_ = cot α_d_	to include simple sloped walls

### 3.3 Hyperparameter tuning

Hyperparameter refers to user-accessible parameters in ML algorithms that can be adjusted to tailor the model to a specific dataset. Unlike model parameters, which are determined by the algorithm itself (e.g., kernel functions in SVR), hyperparameters are manually set by the user to optimize model performance. Proper tuning of hyperparameters ensures efficient computational resource use, enhances model accuracy, and reduces the risk of overfitting.

In this study, the hyperparameters of the ML models were optimized using Python’s open-source scikit-learn library [58]. Table 1 summarizes the optimal hyperparameters adopted for the SVR, RF, GBDT, and ANN models.

For instance, the term ‘C,’ referred to as the regularization parameter in the SVR algorithm, controls the trade-off between achieving low training error and maintaining model generalization. The range of typical values for this parameter, along with the optimal values identified in this study, is presented in **Table 1**. In ML algorithms, the kernel function plays a critical role by mapping input variables (independent variables) into higher-dimensional spaces to address nonlinearity in the data. Among the kernel functions tested in this study, linear, polynomial, and radial basis function (RBF) kernels were iteratively evaluated to determine the best fit for the dataset. To optimise hyperparameters, this study employed *RandomizedSearch* combined with k-fold cross validation (CV). CV is a widely recognised method for training ML models and ensuring robust validation. It involves dividing the dataset into multiple subsets (folds), performing sampling and re-sampling to eliminate biases in the prediction model [58,59], and reducing the risk of overfitting.

In k-fold CV, the dataset is partitioned into k equal-sized folds. During each iteration, one-fold is reserved as the validation set, while the remaining folds are used for training. This process is repeated k times, ensuring that each fold is used for validation exactly once. The results from all iterations are aggregated to evaluate model performance comprehensively. Initially, the algorithm trains and validates the dataset using CV, with the goal of identifying the optimal hyperparameter set. Once the best hyperparameters are determined, the model is tested on an independent dataset to assess its performance on unseen data. Validation ensures that the model is thoroughly trained on the dataset without overfitting to specific patterns or trends, enabling it to generalize effectively.

**Fig 3:**
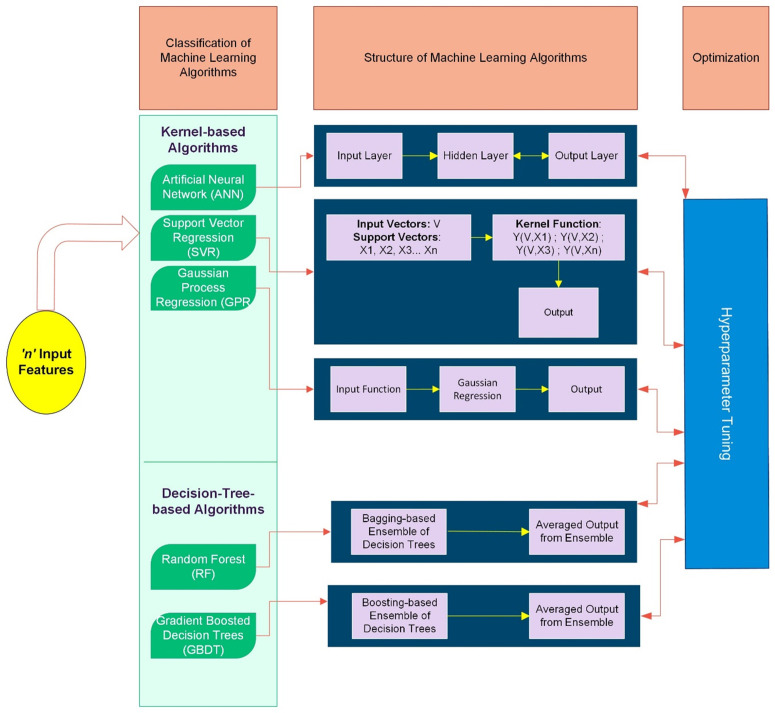
Structure and the optimization technique of the algorithms adopted in the study.

The structure of the algorithms along with the optimization measures introduced by hyperparameter tuning are summarized in Fig 3.

### 3.4 Evaluation metrics

The performance of the ML algorithms in predicting the mean overtopping rates was assessed using comprehensive range of statistical metrics, namely, coefficient of determination (*R*^*2*^), the Root Mean Square Error (RMSE) and Mean Absolute Error (MAE). The coefficient of determination *R*^*2*^ represents the proportion of variance in the dependent variable that is explained by the independent variables. It is calculated using Eq (12).


$$R^{\text{2}}=\text{1}-\frac{\sum ({y_{i}-{\hat{y}}_{i})}^{\text{2}}}{\sum {{(y}_{i^{-}}\overset{―}{y})}^{\text{2}}}$$ (12)

where $y_{i},\;{\hat{y}}_{i}\;and\;\overset{―}{y}\;$represent the observed values, predicted values, and mean of all observed values, respectively.

The Root Mean Square Error (*RMSE*) gauges the standard deviations between the observed and predicted values and the Mean Absolute Error (*MAE)* can be used to express the discrepancies between the observed and predicted values averaged over the total number of observations. The *RMSE* and *MAE* were computed using Eqs (13) and (14), respectively.


$$\begin{array}{c} \text{RMSE}\;=\sqrt{\frac{\text{1}}{N}\sum {\mathrm{\backslash nolimits}}_{i=\text{1}}^{N}{\left(y_{i}-{\hat{y}}_{i}\right)}^{\text{2}}} \end{array}$$ (13)

$$\begin{array}{c} \text{MAE}\;=\;\frac{\text{1}}{N}\sum {\mathrm{\backslash nolimits}}_{\text{1}}^{N}\left|y_{i}-{\hat{y}}_{i}\right| \end{array}$$ (14)

where$\;y_{i}$ and ${\hat{y}}_{i}$ are the actual and predicted values, respectively.

The statistical significance of the predictions generated by the ML models was evaluated using the *F-test.* In regression analysis, the null hypothesis assumes that the model is non-predictive, implying that all regression coefficients are zero. The *F-test* score determines the acceptance or rejection of the null hypothesis. The assessment of the statistical significance is based on the improvement of regression models when predictor variables are iteratively added to the model from a starting point of zero predictor variables. The *F-test* score can be determined using Eq (15) and is the ratio of the explained variance to the unexplained variance of the regression model.


$$F=\;\frac{\frac{SSR}{k}}{\frac{SSE}{n-k-\text{1}}}$$ (15)

where Sum of Squares, Regression, $SSR=\;\sum ({y_{i}-{\hat{y}}_{i})}^{\text{2}}$, Sum of Squares, Error $SSE=\;\sum {{(y}_{i^{-}}\overset{―}{y})}^{\text{2}}$, *k* and *n* are the numbers of independent variables and observations, respectively.

### 3.5 Feature importance analysis

The contribution of individual features in predicting overtopping quantities using ML algorithms was analyzed through Shapley values, commonly known as the SHAP index. Originally developed to measure the importance of individual players in a team [60], SHAP indices have since been adapted for interpreting ML models.

As ML applications have advanced, the need to interpret model outcomes beyond raw predictions has grown. A SHAP-index analysis involves several steps. First, a reference model is developed using all features in the dataset, and its performance is evaluated. Next, a ‘permutation and combination’ approach are applied to generate all feature combinations. Shapley values are calculated by assessing the incremental contribution of individual features to the prediction task. These values can be positive or negative, with higher SHAP values indicating stronger feature influence and lower values indicating weaker influence. A graphical representation of SHAP indices highlights the importance of individual features in the prediction process. This analysis supports informed decision-making by providing deeper insights into the outcome of predictive ML models [61].

### 3.6 Database

**Fig 4:**
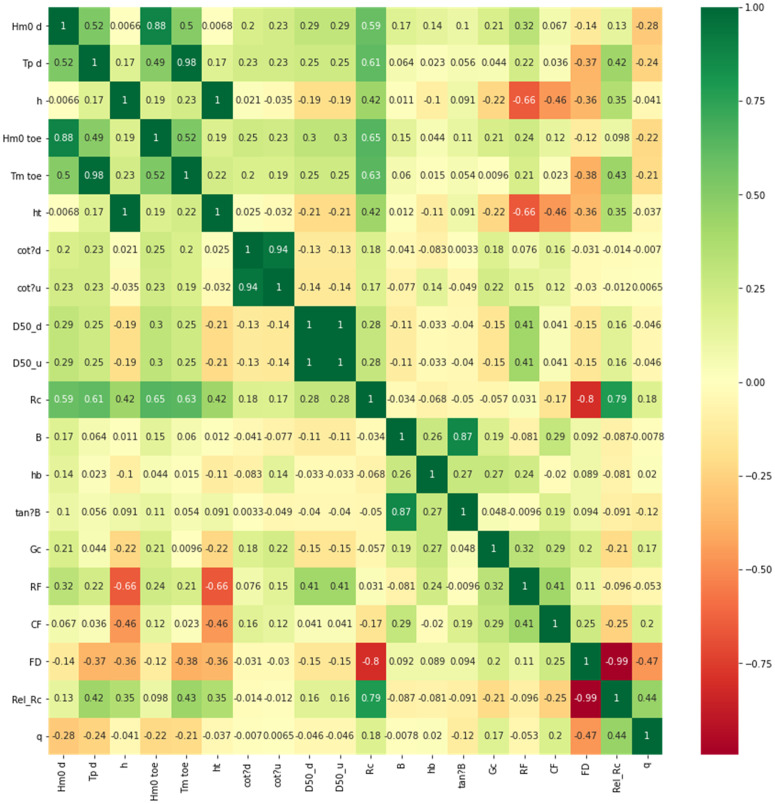
Correlation heatmap of the different overtopping parameters in the dataset.

The EurOtop database [7] serves as a comprehensive guideline for designing and assessment of coastal defence structures such as sloping structures (e.g., seawalls and breakwaters). It comprises a collection of mean overtopping rates obtained from comprehensive physical modelling tests. In this study, 1078 tests of mean overtopping rates at sloping structures were filtered from the 8) database according to the following criteria shown in Table 2:

The main dataset [7] contained 33 features (dependent variables), with the mean overtopping rate (*q*) as the dependent variable. The correlation between the dependent and the independent variables was extremely low, suggesting a non-linear relationship (see heatmap in Fig 4. A sizeable number of parameters showed correlation between 0 to 0.25.

**Fig 5:**
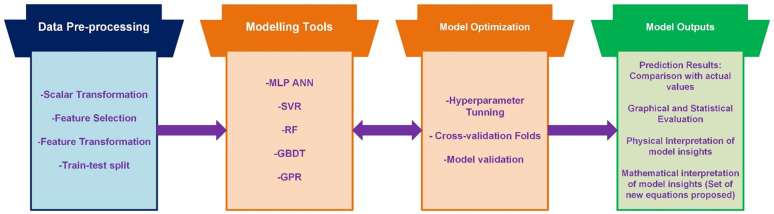
Methodological framework of the study.

ML algorithms investigated in this study can handle such nonlinear relationships effectively. To minimise data redundancy and focus on the most influential features for predicting mean overtopping rates, pre-processing steps such as feature selection and transformation were applied, as discussed in Section 3.2. After these steps, the processed dataset consisted of 19 key features, which are presented in Table 3, along with their respective value ranges. A common methodological framework was adopted to ensure a rigorous analysis (Fig 5). The dataset was split into 70% for training and 30% for testing the ML models. Following the analysis, the predicted mean overtopping rates from the test-set were compared to the actual values in the EurOtop dataset. Optimization techniques, such as hyperparameter tuning and cross-validation, were employed to ensure robust and accurate predictions. The output of the models included the predicted mean overtopping rates and a feature importance analysis, which identified the most influential features for the prediction task. The accuracy of the predictions was assessed using standard statistical metrics, and physical and mathematical interpretations were conducted to extract meaningful insights from the models.

**Table 3:** List and range of overtopping parameters included in the analysis.

Feature	Description	Range of values in dataset
H_m0d_	Significant wave-height in deep water [m]	0.02 to 0.3
T_pd_	Time period of incident waves in deep water [s]	0.85 to 3.56
h	Water depth in front of the structure [m]	0.11 to 0.75
H_m0,toe_	Significant wave-height at toe of the structure [m]	0.02 to 0.32
T_m,toe_	Time Period at toe of the structure [s]	0.77 to 3.56
h_t_	Water depth in front of the structure [m]	0.07 to 0.75
cotα_d_	Cotangent of angle between structure slope downward berm and horizontal [-]	1.33 to 2
cotα_u_	Cotangent of angle between structure slope upward berm and horizontal [-]	1.33 to 2
D_50,d_	Nominal diameter of rock, downward slope [m]	0.026 to 0.1
D_50,u_	Nominal diameter of rock, upward slope [m]	0.026 to 0.1
R_c_	Crest freeboard [m]	0.001 to 0.37
B	Berm width [m]	0
γ_f_	Permeability and Roughness Factor [-]	0.38 to 0.59; ≠ 0.55
tanα_B_	Tangent of angle that sloping berm makes with horizontal [-]	0
G_c_	Width of promenade [m]	0 to 0.875
RF	Reliability Factor [-]	1–3
CF	Complexity Factor [-]	1–3
FD	Freeboard Deficit [-]	0.5 to 1
Rc/H_m0,toe_	Relative Freeboard [-]	0 to 2.83

## 4.0 Results and discussion

This section elaborates the performance of the ML models in predicting mean overtopping rates and measures applied to interpret the findings from such models. Based on the results from the ML models, an equation is developed to interpret the ML models into mathematical terms.

### 4.1 Model performance

**Fig 6:**
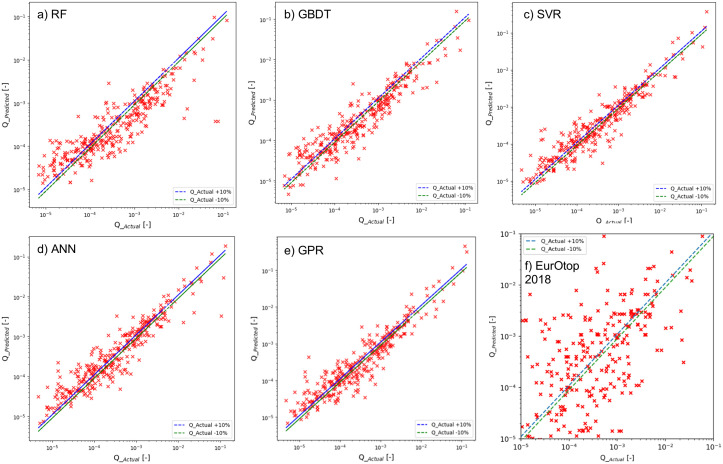
Graphs showing comparison of Q__Predicted_ and Q__Actual_. a) RF, b) GBDT, c) SVR, d) ANN, and f) EurOtop (2018).

The dataset obtained from [7] was filtered for entries related to simple sloped breakwaters using a set of criterion and then pre-processed and curated in advance. Pre-processing steps included, scaler transformation, imputation of missing values by interpolation (due to ANN’s inability to analyse data with missing values), feature selection and transformation. Following the pre-processing steps the data were inputted into the ML algorithms and hyperparameter tuning was conducted to ensure that the algorithms were optimized for the input data to save computational time and resources. The models after having trained on the data, yielded predictions from the test set. The predicted estimates of mean overtopping rates were converted to a dimensionless quantity ‘Q__predicted_’ (= $\frac{\text{q}\_\text{predicted}}{\sqrt{\text{9}.\text{81}*({{\text{H}}_{\text{m},\text{toe}})}^{\text{3}}}}$) which is based on the raw values of the predicted mean overtopping rates and the significant wave height at the toe of the structure. Similarly, the actual overtopping rates in the training set were also converted to a dimensionless quantity ‘Q__actual_’ (=$\frac{\text{q}\_\text{actual}}{\sqrt{\text{9}.\text{81}*({{\text{H}}_{\text{m},\text{toe}})}^{\text{3}}}}$). The comparison of Q_predicted versus Q_actual is depicted in Fig 6. Graphical analysis is suggestive of the fact that for smaller overtopping quantities all the algorithms exhibited reasonable accuracy between ‘Q__predicted_’ and ‘Q__actual_.’ The nature of the graphical distribution of the actual and predicted values were consistent across all the algorithms that highlighted the consistency of the methods. The spread of larger overtopping quantities from the 95% confidence interval can be explained from the fact that instances of such quantities were somehow less than smaller overtopping quantities in the dataset. The ML algorithms are data-driven techniques and hence the scarcity of larger overtopping quantities should influence the training of the algorithms and subsequently affect the performance of data predictions. It could also be inferred from the positioning of datapoints in Fig 6(f) that, all the ML algorithms yielded better performance than the existing EurOtop (2018) equations (i.e., Eqn1 and Eqn 2).

The prediction performance of the algorithms and the observed patterns were similar to recent studies such as Habib et al. [62]. The graphical analysis of the performance of the ML models can further be investigated in numerical terms using the evaluation metrics shown in Table 4. The evaluation metrics revealed that the algorithms performed like one another. Strong *r*^*2*^ scores were exhibited by all the models (*r*^*2*^* *> 0.40; [63]) that is suggestive of the fact that there was reasonable agreement between the predicted and actual data. Furthermore, the ANOVA *F-test* was performed to investigate the statistical significance of the prediction models. The scores from the *F-test* for all the models were significantly higher than the critical value of 3.87 together with extremely low *p-values* at a significance level of 0.05. Results from the *F-test* indicated that the dependent variable (here, the predicted mean overtopping rates) were able to explain the variance in the independent variables (here, the actual mean overtopping rates). The extent of outliers in the prediction models were investigated by the RMSE score. Higher *r*^*2*^ values were complemented with lower RMSE values and vice versa meaning that models exhibiting better agreement between the actual and predicted mean overtopping rates should have lower RMSE values. The RMSE values varied between 0.100 to 0.685 among the models with GPR yielding the highest RMSE and ANN the lowest. Here, it was evident form the statistical metrics that kernel-based methods yielded better predictions compared to the DT based methods, RF and GBDT. As evident from the statistical metrics, the ML algorithms exhibited better prediction performance than the existing EurOtop (2018) equations.

**Table 4:** Statistical analysis of the prediction results from the ML algorithms.

Algorithm	Coefficient of determination- R^2^	Root mean square error-RMSE	Mean absolute error (MAE)	Relative absolute error (RAE)	*ANOVA F-test* score
RF	0.61	0.685	0.163	0.30	389
GBDT	0.63	0.640	0.156	0.30	593
SVR	0.70	0.568	0.147	0.28	789
ANN	0.59	0.685	0.133	0.36	388
GPR	0.80	0.100	0.013	0.30	1080
EurOtop (2018)	0.36	0.714	0.221	0.46	265

### 4.2 Feature importance and physical interpretation

**Fig 7:**
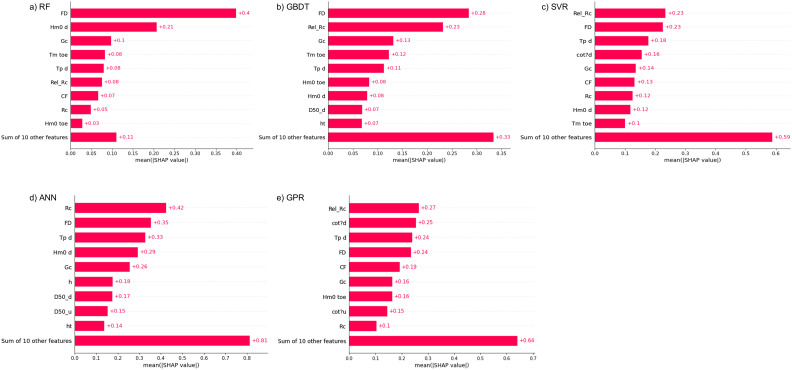
Feature Importance Analysis of the Algorithms expressed by SHAP indices. a) RF, b) GBDT, c) SVR, d) ANN, and e) GPR.

The SHAP indices shown in Fig 7 revealed the importance of the individual features in the prediction task. It is important that ML models identify similar features as the most important ones as this result indicates the consistency of the algorithms. Freeboard Deficit, FD, was consistently identified as one of the top three features across all the algorithms. This fact agrees with the physical phenomenon of the overtopping process where overtopping should be influenced by FD. The study of [9] reported that FD explains the overtopping phenomenon more explicitly than freeboard and relative freeboard as it considers wave run-up assessment. Here also, in the feature importance analysis, the importance of FD is evident. The results indicated that although the ML-algorithms are data-driven techniques, the physics of the overtopping phenomenon was highlighted consistently across all the algorithms.

**Fig 8:**
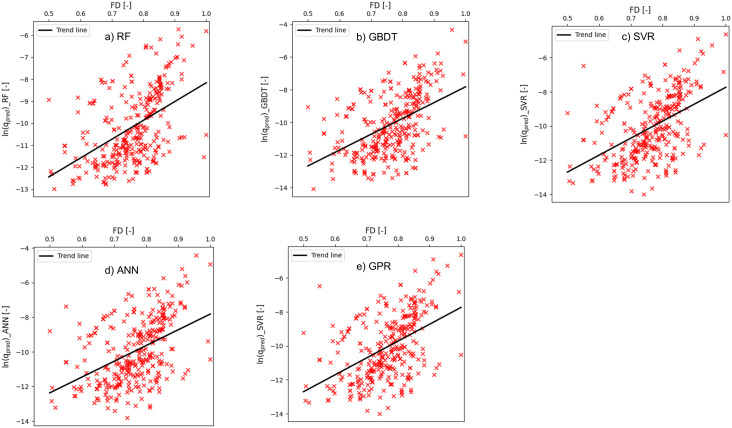
Variation of ln(q_pred_) with FD. a) RF, b) GBDT, c) SVR, d) ANN, and e) GPR.

Further investigation into the relationship between FD and the logarithmic value of predicted overtopping rates was conducted and the results are illustrated in Fig 8. Relevant studies [64,65] have highlighted that foreshore slope influences wave overtopping to a considerable extent. Therefore, it was important to include the effect of foreshore slope in the overtopping analysis using ML-algorithms. The feature FD accounts for the effect of foreshore slope, structural slope, local wave height and period [9]. The study [9] also reported that although relative freeboard $\frac{R_{c}}{H_{m\text{0},toe}}$ has been effectively used to deduce overtopping analysis, using the term FD adds the dimensions of local wave conditions and the foreshore slope to the overtopping analysis. Therefore, it renders FD as a better explanatory variable than relative freeboard in determining overtopping quantities. From Fig 8, it is evident that FD exhibits a strong inverse relationship with the overtopping quantities, and this finding agrees with recent studies where FD was considered a more prominent variable in overtopping estimation.

**Fig 9:**
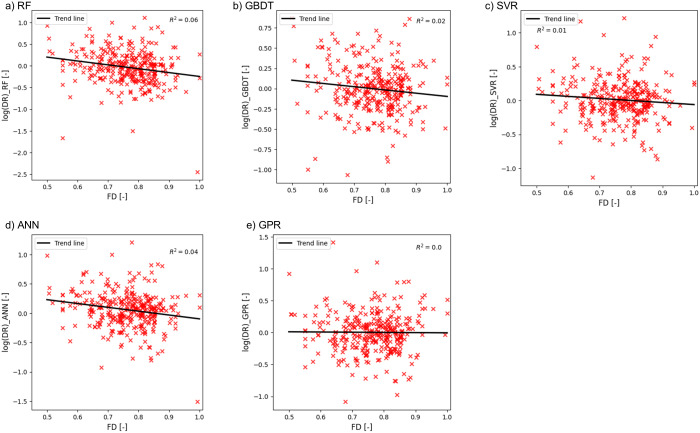
Plot of DR vs FD. a) RF, b) GBDT, c) SVR, d) ANN, and e) GPR.

The Discrepancy Ratio (DR) computes the ratio between the predicted and actual quantities. For an ideal prediction model, the DR should be independent of the key features involved in the prediction task, i.e.,: there should be zero to very less amount of statistical significance between them [66]. The plot of DR versus FD (the overall important feature in the ML models) indicated there was no statistical significance in the relationship between the two variables (see Fig 9). Therefore, the robustness of the predicted overtopping quantities from the ML models could be confirmed.

## 5.0 Mathematical interpretation

The mathematical interpretation of the results of the ML-models was performed in three steps.

i. As a starting point, regression analysis was performed between the overall impactful feature and ln(q) using the *test-set* of the best performing algorithm.
ii. The equation formed in (i) was refined using GP and a synthetic dataset of the feature and ln(q). Both supervised and unsupervised methods were implemented and hence two equations were developed.
iii. The relationship between the most impactful feature and ln(q) was observed in EurOtop dataset. Afterwards, the newly formed equations in (ii) were used to simplify and improve this relationship using FD values from the EurOtop dataset. The resulting simplified relationship would allow a rapid initial assessment of the mean wave overtopping rate using just a single variable.

Findings from the analysis of the ML models revealed that GPR performed the best overall and that FD has a strong relationship with the mean overtopping rate across all the algorithms. In this section, the relationship between FD and the logarithmic value of mean overtopping rates, ln(q), is further investigated to delineate a set of equations. The *test-set* of the GPR algorithm was extracted and FD was fitted with predicted values of mean overtopping rate in the logarithmic form. The fitting yielded the results shown in Table 5.

**Table 5:** Regression results of ln(q) versus FD from the GPR *test-set.*

Regression Type	*R*^*2*^ score
Linear	**0.30**
Logarithmic	**0.27**
Exponential	**0.28**
Polynomial (degree = 2)	**0.40**

Results from **Table 5** indicated that a polynomial expression best represented the relationship between FD and ln(q). The polynomial expression is shown in Eq (16).


$$\text{ln}(q)=\text{4}\text{0}.\text{9}\text{5}*(FD{)}^{\text{2}}-\text{5}\text{0}.\text{2}\text{2}*(FD)+\text{4}.\text{0}\text{3}$$ (16)

The polynomial expression is then fitted on a synthetic dataset of 2000 sets of FD and ln(q) values considering the range of FD from 0.5 to 1. This range of FD was selected based on the EurOtop dataset so that the fitted equation could be applied to the full range of the EurOtop dataset. Afterwards, Eq 17 was modified using Genetic Programming (GP) algorithm. A GP-algorithm typically begins by building a population of naive random formulas to represent a relationship between known independent variables and their dependent variable targets to predict new data. Each successive generation of programs then evolved from the previous one to undergo ‘genetic mutation’ until an expression is deduced that best represents the population (dependent and independent variables). Effectively Eq (16) was modified to Eqs (17) and (18). The difference between Eqs (17) and (18) was that the former was based on a set of supervised mathematical operators to the algorithm while the latter was based on independent or unsupervised operators.


$$\text{ln}(q)=\left(\text{1}.\text{2}\text{3}\text{1}-{FD}^{\text{2}}\right)(-FD-\text{2}.\text{0}\text{3}\text{1})\{\left(\text{4}.\text{7}\text{4}\text{1}*FD)+\text{1}.\text{9}\text{0}\text{6}\right)\}-\text{0}.\text{4}\text{4}\text{3}$$ (17)

$$\text{ln}(q)=\text{1}\text{6}.\text{9}\text{5}FD\left({FD}^{\text{2}}-\text{1}.\text{0}\text{1}\text{7}\right)-\text{0}.\text{6}\text{7}\text{6}(FD)-\text{4}.\text{2}\text{7}$$ (18)

**Fig 10:**
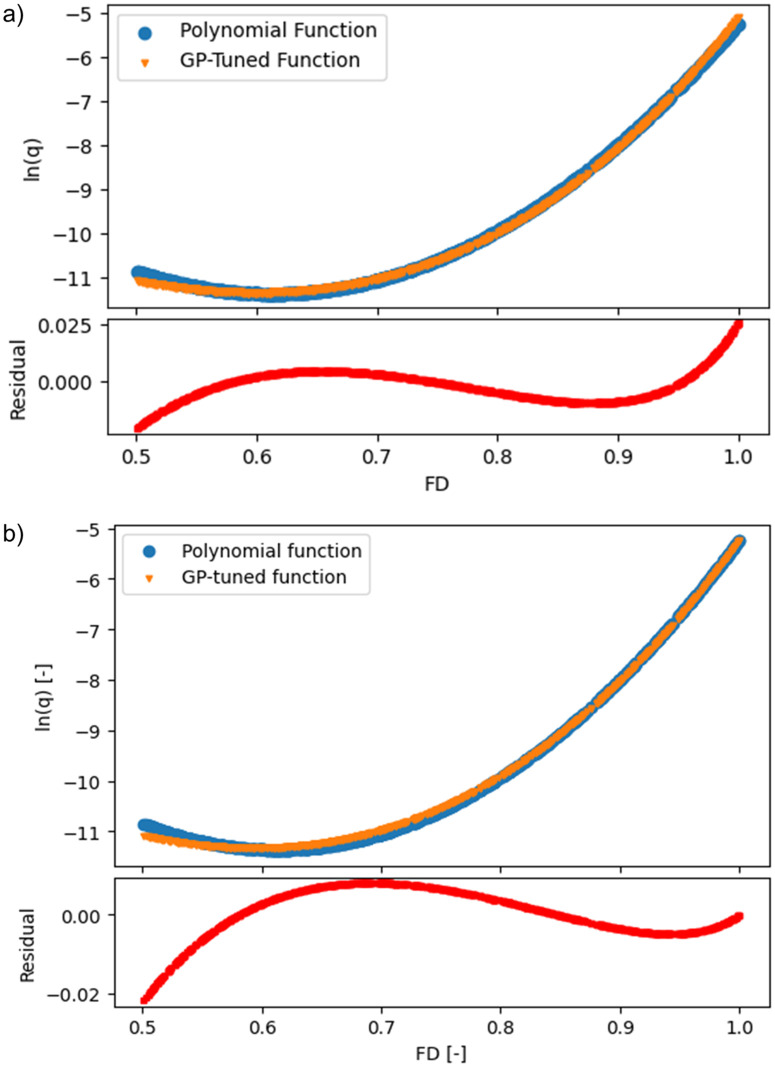
Plot of residuals from a) Eq (17) and b) Eq (18) in predicting ln(q) and their comparisons with Eq 19.

The plot of residuals between the original polynomial equation (Eq 14) and the GP-fitted equations (Eqs. 17 and 18), in predicting ln(q) is shown in Fig 10. The plot of residuals indicated that both Eq (17) and Eq (18) fitted the synthetic data with a significant agreement.

**Fig 11:**
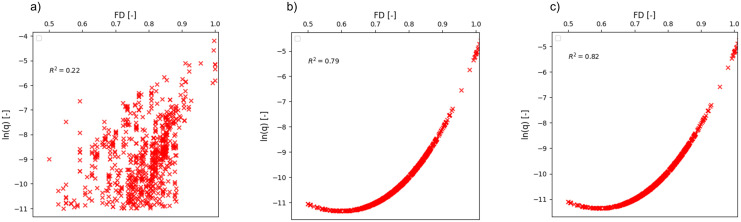
Improvement of the relationship between ln(q) and FD. a) Scatter plot from EurOtop data, and b) Improved scatter plot using Eq 17, and c) Eq 18.

The final phase of the mathematical interpretation involved fitting the Eqs (17) and (16) into the EurOtop data. The objective was to simplify the relationship between FD and ln(q) in the EurOtop data. Simplification of the relationship would result in rapid estimation of ln(q) over the range of FD (0.5 to 1). The scatter plot of ln(q) against FD for the EurOtop data is shown in Fig 11(a). As evident from the Fig 11, there was a significant amount of scatter in the data. Hence, a best-fit graph on this data could result in simplifying the relationship between the two variables. Eqs (17) and (18) were selected for this purpose which was previously fitted on synthetic data. The graphs in Fig 11 (b) and Fig 11 (c) illustrate the values of ln(q) generated from actual values of FD from the EurOtop data. The scatter in the graphs between FD values and ln(q) generated from Eqs (17) and (18) reduced significantly. and also, the *R*^2^ values improved drastically.

Therefore, it could be concluded that the newly developed set of equations were able to improve and simplify the relationship between FD and ln(q) as exhibited in the original EurOtop data.

## 6. Conclusion

Extreme climatic events are intensifying because of climate change, posing significant threats to the structural integrity and reliability of coastal defence structures. In this context, leveraging computational advancements and introducing efficient tools, such as ML-based methods, is crucial for estimating wave overtopping from critical coastal defences and assessing the functionality of these structures and thus enhancing coastal resilience. This study developed a novel methodological framework to examine the mean overtopping rates at sloping coastal defence structures, leveraging advanced ML-based algorithms. A robust methodological framework was developed and tested using five ML algorithms, including RF, GBDT, ANN, SVR, and GPR, for accurate prediction of mean wave overtopping rates from critical sloped coastal defence structures. Physical and mathematical interpretations of the results were performed to enhance user understanding and reproducibility for other datasets.

From both graphical and statistical evaluations, all algorithms demonstrated satisfactory and consistent performance. However, the GPR algorithm outperformed others, achieving the highest *R*^*2*^ value of 0.80 and the lowest RMSE, MAE and RAE values of 0.100, 0.013 and 0.30, respectively. The performance variation across the algorithms was minimal, indicating consistency in predicting mean overtopping rates. Kernel-based models were faster than decision-tree-based models, except for ANN.

Feature importance analysis highlighted FD (dimensionless freeboard) as a key predictor across all models. For four of the five algorithms, FD ranked among the top three most influential features. Consistent with prior studies, FD demonstrated advantages over relative freeboard by incorporating local wave conditions, thereby better representing the physics of overtopping. The predicted mean overtopping rates showed an inverse relationship with FD, aligning with physical expectations.

To further enhance the interpretability of the ML models, a new set of equations was developed based on model insights. Initially, a polynomial relationship was derived to represent the logarithmic mean overtopping rates as a function of FD. This equation was refined using a Genetic Programming (GP) algorithm and a synthetic dataset of FD values (ranging from 0.5 to 1, based on EurOtop data). GP, which has traditionally been used to refine empirical formulae or develop equations from numerical analyses, was uniquely applied here to interpret ML model outputs. The refined polynomial equation was validated with the EurOtop dataset, showing significant improvements in capturing the relationship between FD and logarithmic mean overtopping rates. Despite their effectiveness, ML models struggled to predict large overtopping rates compared to smaller rates. This limitation arises from the imbalance in the EurOtop dataset, which contained more instances of small overtopping rates. Enhancing the models’ performance for large overtopping rates would require datasets with a more balanced representation of both small and large overtopping events.

Overall, the findings demonstrate the potential of ML algorithms as rapid assessment tools for predicting mean wave overtopping rates. These predictions, supported by physical and mathematical interpretations, offer a complementary approach to existing numerical and experimental methods. The equations developed in this study are not intended to replace existing approaches but to supplement them, with the expectation that additional experimental studies and numerical validations will further enhance the robustness of ML-based predictions. This study also highlights several avenues for future research. First, the limited representation of extreme overtopping cases in the dataset constrained the robustness of the ML models, particularly in accurately predicting large overtopping discharge incidences. Expanding the dataset to include more extreme events would improve model generalizability. Second, the dynamic nature of wave overtopping, especially under changing climate conditions, warrants further investigation to account for non-stationary boundary conditions and evolving risk profiles. Finally, incorporating uncertainty quantification into ML-based prediction frameworks remains a critical next step. This advancement would improve model transparency and reliability, thereby strengthening their applicability for risk-informed coastal design and decision-making.
